# Myrsinane-Type Diterpenes: A Comprehensive Review on Structural Diversity, Chemistry and Biological Activities

**DOI:** 10.3390/ijms25010147

**Published:** 2023-12-21

**Authors:** Eduarda Mendes, Cátia Ramalhete, Noélia Duarte

**Affiliations:** 1 Research Institute for Medicines (iMED.Ulisboa), Faculdade de Farmácia, Universidade de Lisboa, Av. Prof. Gama Pinto, 1649-003 Lisboa, Portugal; ermendes@ff.ulisboa.pt (E.M.); catiar@uatlantica.pt (C.R.); 2 ATLÂNTICA—Instituto Universitário, Fábrica da Pólvora de Barcarena, 2730-036 Barcarena, Portugal

**Keywords:** myrsinane, premyrsinane, cyclomyrsinane, polycyclic diterpenes, *Euphorbia*, natural active compounds, biological activity

## Abstract

*Euphorbia* species are important sources of polycyclic and macrocyclic diterpenes, which have been the focus of natural-product-based drug research due to their relevant biological properties, including anticancer, multidrug resistance reversal, antiviral, and anti-inflammatory activities. Premyrsinane, cyclomyrsinane, and myrsinane diterpenes are generally and collectively designated as myrsinane-type diterpenes. These compounds are derived from the macrocyclic lathyrane structure and are characterized by having highly oxygenated rearranged polycyclic systems. This review aims to describe and summarize the distribution and diversity of 220 myrsinane-type diterpenes isolated in the last four decades from about 20 *Euphorbia* species. Some myrsinane diterpenes obtained from *Jatropha curcas* are also described. Discussion on their plausible biosynthetic pathways is presented, as well as isolation procedures and structural elucidation using nuclear magnetic resonance spectroscopy. Furthermore, the most important biological activities are highlighted, which include cytotoxic and immunomodulatory activities, the modulation of efflux pumps, the neuroprotective effects, and the inhibition of enzymes such as urease, HIV-1 reverse transcriptase, and prolyl endopeptidase, among other biological effects.

## 1. Introduction

Natural products (NPs) have been crucial in drug discovery and development, with more than half of the current clinically used drugs based on natural compound pharmacophore [1]. In fact, over the last four decades, nearly two-thirds of medications containing novel chemical entities have come from NPs or chemically created analogues of NPs [2], highlighting their role in the treatment of cancer and infective diseases but also cardiovascular diseases and even diabetes and multiple sclerosis [3]. Some relevant examples include the antidiabetic drugs liraglutide and semaglutide, which result from the optimization of the exendin-4 peptide obtained from Heloderma lizard venom [4]; fingolimod, a derivative of the natural metabolite myriocin isolated from *Isaria sinclairii* used as a sphingosine-1-phosphate (S1P) receptor modulator to treat multiple sclerosis [5,6]; and even, paclitaxel (Taxol) isolated first from the Pacific yew *Taxus brevifolia* and currently used to treat a high variety of cancers [7,8]. These molecules can be used directly in therapy as they are isolated from natural sources or obtained through chemical processes, creating derivatives or even fully synthetic compounds, which use the natural scaffold as a structural model to create more effective analogues [9].

The importance of using natural compounds as medicines is related to their wide range of recognized biological activities and unique structural features, which can be extremely useful to overcome drug–target and protein–protein interaction issues [10]. In fact, when compared to traditional synthetic medicines, natural compounds have unique properties, such as higher molecular weights, more sp^3^ carbon and oxygen atoms, less nitrogen and halogen atoms, and more H-bond acceptors and donors, among other features [3]. Although pharmaceutical companies’ efforts to discover new natural products have significantly decreased over recent decades, the values presented by Newman and Crag (2020) demonstrated that the “influence of natural product structures” has not significantly decreased in that time, at least with regard to drug approvals that are based on such structures [2,3,11].

The *Euphorbia* genus comprises more than 2000 recognized species, being the largest genus of the Euphorbiaceae family and the third largest genus of angiosperm plants. *Euphorbia* species have a worldwide distribution, exhibiting an extraordinary variety in morphology, from small ephemeral, annual, or perennial herbaceous plants to big shrubs, small trees, lianas, and even cactus-like succulents [12,13]. Many *Euphorbia* species have been historically used to cure digestive and respiratory problems, inflammation, intestinal parasites, and tumors, among other conditions [12]. These therapeutic properties are attributable to the presence of different secondary metabolites, namely, polycyclic and macrocyclic diterpenes (lathyranes, jatrophanes, and their rearranged derivatives), among others. Diterpenoids have been the focus of natural-product-based drug development among *Euphorbia* species due to their broad spectrum of therapeutically relevant bioactivities, including anticancer, multidrug resistance reversal, antiviral, and anti-inflammatory activities [12]. For example, an ingenane ester from *E. peplus*, ingenol 3-angelate (ingenol mebutate, PEP005, Picato^®^, LEO Pharma, Ballerup, Denmark), was authorized by the FDA in 2012 and the EMA in 2013 to treat actinic keratoses. However, due to negative side effects, this medication has sadly been discontinued [14]. On the other hand, resiniferatoxin, a daphnane orthoester derived from *E. resinifera*, is a strong capsaicin receptor agonist that is now being tested in a phase III clinical study for the treatment of overactive bladders and chronic pain [12].

Over the last two decades, several reviews have been published reporting the isolation and biological activities of the different classes of diterpenes from *Euphorbia* species. Some of these reviews covered all the diterpene classes [12,13,15,16,17,18,19,20], while others focused on a specific class of compounds, namely, ingenane [21], daphnane [22], jatrophane [23], or lathyrane diterpenoids [24]. There is also a review describing diterpenes containing a *gem*-dimethylcyclopropane [25], one that reported all the diterpenes isolated from *E. fischeriana* Steud [26], and another focused on diterpenes with a specific activity [27]. 

To the best of our knowledge, to date, there are no reviews exclusively addressing myrsinane-type diterpenes. Therefore, this comprehensive review will focus on premyrsinane-, cyclomyrsinane-, and myrsinane-type diterpenes, covering the period from 1995 to 2023, with emphasis on their biosynthesis, isolation, structural identification, and biological activities.

The literature search was carried out from January to April 2023 on Web of Science, ScienceDirect, and PubMed, using several combinations of keywords and truncation (e.g., combinations of myrsinane, premyrsinane, and cyclomyrsinane with *Euphorbia*, *Jatropha*, and biological activity). The timespan from 1995 to 2023 was considered. Only peer-reviewed research articles or reviews were considered; no restrictions on language or the geographical origin of authors were applied. The literature was individually screened by the authors, applying as exclusion criteria, poor quality, inaccurate data, and articles or reviews that were not considered relevant to the aim of the review. Mendeley Reference Manager Software (version 1.19.8, 2020) was used to manage the references and eliminate duplicates.

## 2. Phytochemistry

### 2.1. Biosynthetic Considerations 

Terpenes, also known as isoprenoids, are, by far, the most diverse group of secondary metabolites, comprising over 80,000 identified natural compounds. They have a wide distribution in plants, microorganisms, insects, and marine invertebrates, playing a crucial role in the metabolism and gathering of an important source of bioactive compounds [28,29]. The terpenic scaffold results from the assembly of the C5 units of isopentenyl diphosphate (IPP) and its isomer dimethylally pyrophosphate (DMAPP) from two major biosynthetic routes, the mevalonate and the 2-C-methyl-d-erythritol-4-phosphate pathways. The variation in the number of repeats, rearrangements, and cyclization reactions catalyzed by prenyltransferases and terpene synthases gives rise to the chemical and structural diversity. Accordingly, they are classified as hemiterpenes (C_5_), monoterpenes (C_10_), diterpenes (C_20_), triterpenes (C_30_), and tetraterpenes (C_40_) based on the number of the five carbon units.

**Figure 1:**
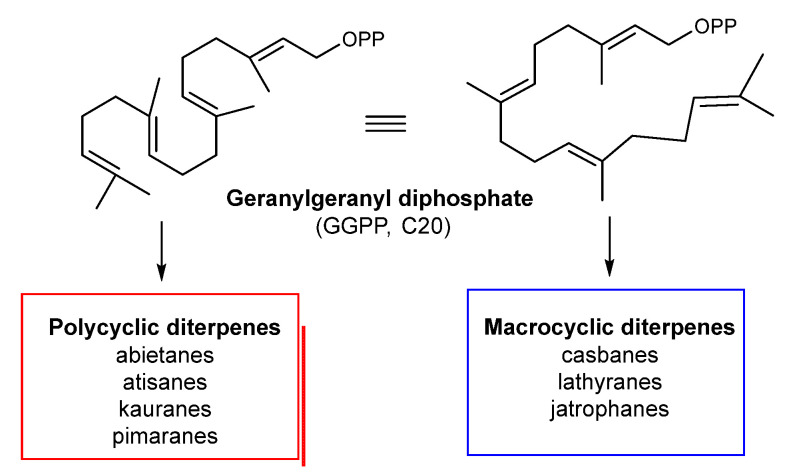
From GGPP to polycyclic and macrocyclic diterpenes—a biosynthetic proposal (adapted from [18]).

Diterpenes arise from geranylgeranyl diphosphate (GGPP, C_20_) via electrophilic cyclization reactions mediated by carbocation formation and catalyzed by diterpene cyclases and further Wagner–Meerwein rearrangements, which generate their vast chemical diversity [30,31,32]. One of the most important cyclization reactions of GGPP is the formation of copalyl diphosphate (copalyl-PP), which plays a central role in the biosynthesis of polycyclic diterpenes, such as abietanes, pimaranes, kauranes, and atisanes (Figure 1) [32]. The second mode of GGPP cyclization leads to macrocyclic structures, namely, the monocyclic cembrane, the bicyclic casbane, and its unsaturated derivative, casbene. Even though the biogenetic routes are poorly understood and many mechanistic details remain uncertain, these diterpenes have been considered the precursors of lathyrane and jatrophane frameworks [23,32,33,34]. These structurally unique polyoxygenated macrocyclic diterpenes and their polycyclic derivatives have a very limited distribution in nature, being restricted to the Euphorbiaceae and Thymelaceae families, which increases their biogenetic and chemotaxonomic significance [12,15,27,35]. Over the last two decades, macrocyclic diterpenes and their derivatives have been the subject of academic attention due to their biogenetic relevance, structural complexity, and biological properties [12,15,20,23].

**Figure 2:**
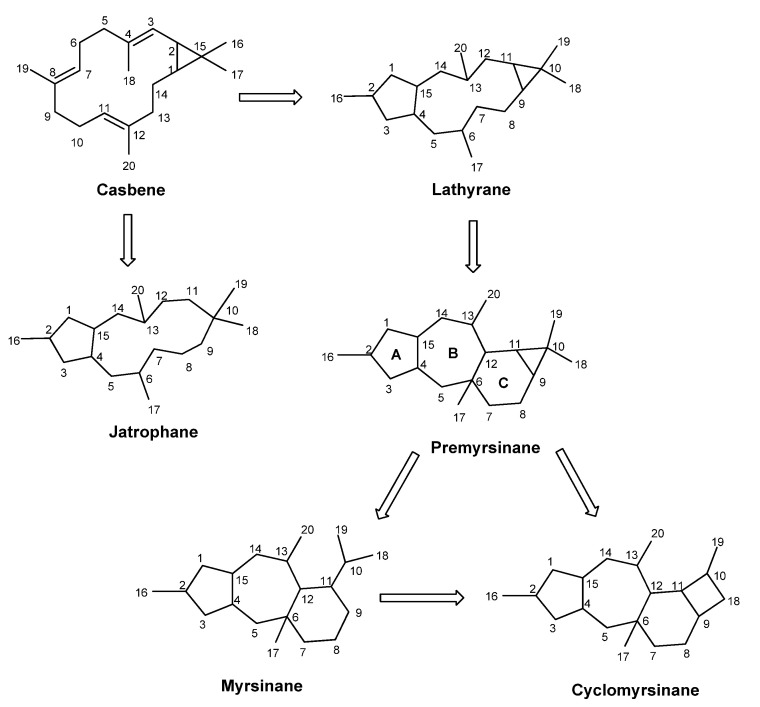
Biosynthesis of jatrophane-, lathyrane-, and myrsinane-type diterpenes.

Lathyrane diterpenes are characterized by a flexible twenty carbon skeleton, with a 5:11:3 fused ring system derived from the intramolecular cyclization of casbene [31,33]. Further intramolecular reactions and rearrangements on the lathyrane framework create several polycyclic derivatives, including the jatropholane-, tigliane-, ingol-, and premyrsinane-type diterpenes (Figure 2). Premyrsinanes, myrsinanes, and cyclomyrsinanes are generally and collectively designated as myrsinane-type diterpenes. Only a few explanations concerning the biosynthesis of this kind of diterpenes have been reported, and the subject is still controversial [18,36,37,38]. Accordingly, it is suggested that premyrsinanes derive from the cyclization at C-12 and C-6 of lathyranes, giving rise to the 5/7/6/3-fused tetracyclic skeleton. Myrsinane structures derive from the cleavage between C-9 and C-10, forming a 5/7/6-fused tricyclic structure. On the other hand, cyclomyrsinane-type diterpenes result from a C-C bond migration to create the cyclobutane ring characteristic of these compounds. Similar to the lathyranes, myrsinane-type diterpenes also possess a high variety of oxygenated functions (hydroxyl, keto, epoxy) and acyl groups, such as acetyl, propionyl, butanoyl, isobutanoyl, 2-methylbutanoyl, angeloyl, tigloyl, benzoyl, and nicotinoyl. Due to biogenetic reasons, Rings A and B and Rings B and C have a *trans* configuration, while the cyclopropane ring has a *cis* configuration with H-9α and H-11α. Structural variants also include different stereochemistry on some chiral centers, the number and position of the double bonds, and the frequent presence of a hemiacetal or a 13/17-epoxy ring.

The first myrsinane diterpenes were isolated from *E. myrsinitis* as polyester mixtures and reported in 1982 by Rentzea et al. [39]. In this work, the ester M1 was reduced with LiAlH_4_, yielding the parent polyalcohol as a mixture of epimers (14β:α, 2:1, Figure 3), for which the name myrsinol was proposed, thus giving the name of the entire series [39].

**Figure 4:**
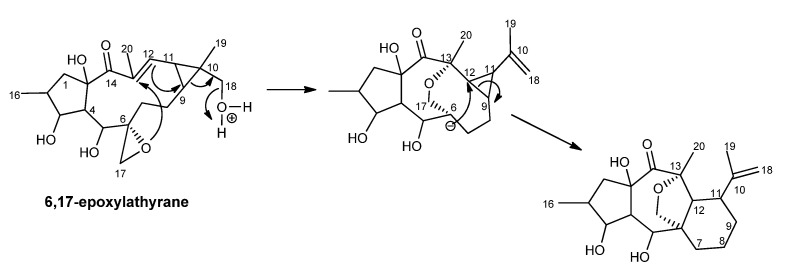
6,17-epoxylathyrane as the myrsinol biosynthetic precursor, as proposed by Rentzea and Hecker [36].

From a biogenetic point of view, the authors postulated that 13,17-epoxymyrsinol would be derived from 6,17-epoxy-18-hydroxylathyrol, as depicted in Figure 4 [36]. More than ten years later, the first premyrsinane-type diterpenes (**154**–**159**) were isolated from *E. allepica* [40,41], and cyclomyrsinol diterpenes (**52**, **53**, **208**) were isolated from *E. prolifera* [42] and *E. seguieriana* [37]. Jeske et al. corrected the stereochemistry at C-5 that had been wrongly assigned as H-5α [37,39]. Therefore, based on the NOE correlations and the existence of a large *J_4_*_,5_ coupling constant value (11.5 Hz) that was indicative of the *trans* orientation of H-4 and H-5, the ester substituent at C-5 was assigned to have an α-orientation [37]. 

**Figure 5:**
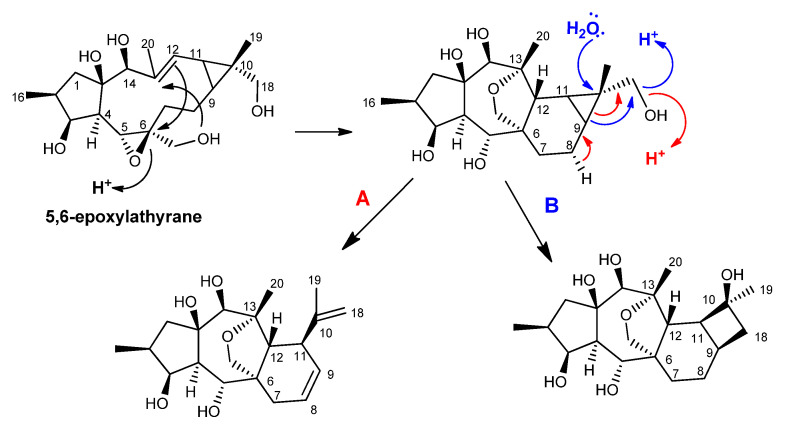
5,6-epoxylathyranes as premyrsinol, myrsinol, and cyclomyrsinol biosynthetic precursors, as proposed by Jeske et al. [37].

A different biogenetic pathway was also suggested, taking 5,6-epoxylathyranes as a precursor (Figure 5) [37]. In this way, the premyrsinane ring system seems to be derived from the opening of the 5,6-epoxy, followed by the formation of the C-6/C-12 bridge. Further protonation of the C-18 hydroxyl group leads to the dehydration and opening of the cyclopropane ring, creating the myrsinane structure (Figure 5—pathway A). On the other hand, the cyclomyrsinane scaffold was suggested to be formed by the nucleophilic attack of a water molecule on C-10, dehydration of the C-18 hydroxyl group, and C-9/C-18 bond migration, giving rise to the cyclobutane ring (Figure 5—pathway B).

**Figure 6:**
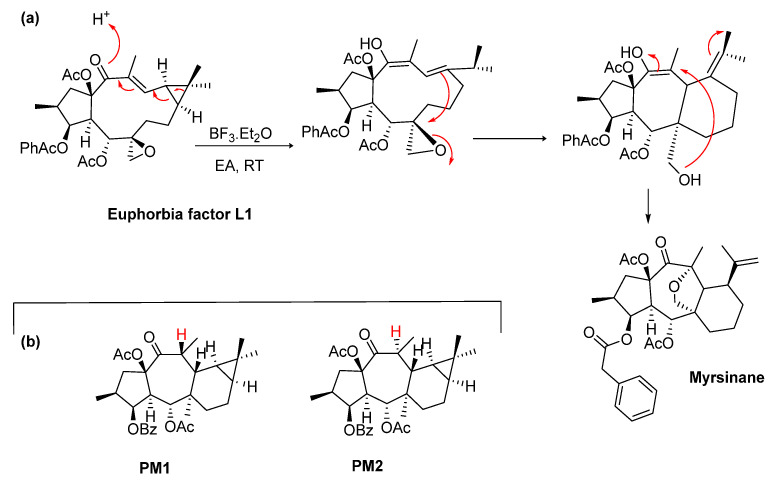
(**a**) Chemical transformation of Euphorbia factor L1 as proposed by Wang and collaborators in 2019 [45]. (**b**) Structure of PM1 and PM2 obtained under the catalysis of iron [46].

Nowadays, the scientific research regarding the biogenetic relationships on lathyrane- and myrsinane-type diterpenes is still very scarce, and only a few studies have been carried out aiming at mimetizing those transformations under laboratory conditions [43,44,45,46]. Recently, Wang et al. proposed a chemical conversion method to obtain two new diterpenes with the myrsinane and euphoractane skeletons from a lathyrane diterpene (Euphorbia factor L1), together with an unnatural diterpene with a 5/7/7/4 fused-scaffold [45]. Starting from the lathyrane-type compound, the postulated mechanism involved a Lewis acid-mediated reaction in which the cyclopropane opening/intramolecular Michael additions are involved to build the various diterpene skeletons, including the myrsinane, as depicted in Figure 6, confirming the biosynthetic connection between these compounds [45]. Another chemical transformation process from lathyranes to premyrsinanes was achieved in a study conducted by Xiao et al. (Figure 6a) [46]. Two new premyrsinane-type diterpenes (PM1 and PM2, Figure 6b), as diastereomers, were synthesized from the Euphorbia factor L3 under the catalysis of iron, involving intramolecular Michael addition reactions with free radicals [46]. 

### 2.2. Isolation and Structural Identification 

The majority of myrsinane-type diterpenes have been isolated from *Euphorbia* species; however, some compounds have also been isolated from *Jatropha* species, in particular *Jatropha curcas* [47,48,49].

These compounds are usually obtained from the dried or fresh whole plant, but other specific parts, such as the seeds, roots, and latex, have also been used [50,51]. The isolation of diterpene polyesters is challenging as they are frequently present in plants only in trace amounts and as complex mixtures of compounds with the same core scaffold. Therefore, a multistep procedure is always necessary. After grinding, the extraction of the vegetable sample is usually carried out via maceration at room temperature using organic solvents, which are critical to ensure the efficient extraction of the target compounds. Commonly used solvents include methanol, ethanol, acetone, chloroform, petroleum ether, and mixtures of these solvents. Due to the ester nature of the compounds, acidic or basic reagents should not be used to avoid hydrolysis or transesterification reactions. Evaporation of the solvent under vacuum at low temperature (35–45 °C) results in a crude extract that needs to be further fractionated. The next step is generally a liquid–liquid partition process, where *n*-hexane or petroleum ether are usually used to defat the extract, followed by a partition with ethyl acetate (EtOAc) and water or mixtures with acetonitrile in order to concentrate the diterpenic compounds in the organic phase. Depending on the extraction solvents and the amount of the crude extract, other techniques could also be used, including vacuum liquid chromatography over RP-18 silica gel or poliamide to remove fats, hydrophilic compounds, and chlorophylls [52,53,54,55]. The EtOAc or acetonitrile crude fraction previously obtained is further fractionated through a combination of different chromatographic techniques, with column chromatography over silica-gel being the first to be carried out. Normally, this first column is eluted with mixtures of *n*-hexane-EtOAc or *n*-hexane-CH_2_Cl_2_ of increasing polarities. NMR or even LC-MS/MS analysis has been frequently used to identify the fractions rich in diterpenes, aimed at conducting a directed and less time-consuming isolation [50,56,57]. The chromatographic fractions are monitored using thin layer chromatography (TLC) and associated based on their similar profile. Not all diterpenes exhibit UV absorption at 254 nm, and there is no specific visualization reagent for their identification. Therefore, the spots must be identified by spraying with suitable visualization reagents, such as mixtures of H_2_SO_4_/acetic acid/H_2_O or H_2_SO_4_/H_2_O (1:1), followed by heating at 105 °C until the development of a black or dark brown color. Further fractionations can be achieved using normal silica gel and other different adsorbents, including reversed-phase silica gel and Sephadex LH-20 gel. In addition, vacuum liquid chromatography (VLC) and centrifugal planar chromatography (CPC) have also been used. Preparative high-performance chromatography, supercritical fluid chromatography (SFC), recrystallization, and preparative thin-layer chromatography are also employed to obtain pure compounds [50,56,57]. 

A combination of extensive spectroscopic techniques including 1D (^1^H- and ^13^C- NMR and DEPT) and 2D nuclear magnetic resonance (^1^H-^1^H-COSY, HSQC, HMBC, and NOESY), mass spectrometry (MS), and infrared (IR) spectroscopy has to be employed for the structural elucidation and identification of the isolated compounds.

For practical reasons, in this review, it would not be possible to present and discuss the NMR data of all the structural variations of myrsinane-type diterpenes isolated to date. Notwithstanding, the ^1^H- and ^13^C-NMR spectroscopic data of some representative premyrsinane, myrsinane, and cyclomyrsinane diterpenes, and the most important features are summarized and discussed below. Myrsinane-type diterpenes rarely occur as free alcohols but rather in the esterified form.

**Figure 7:**
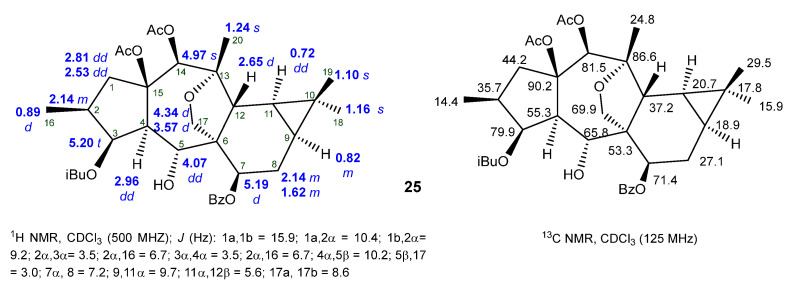
^1^H- and ^13^C-NMR spectroscopic data of the premyrsinane diterpene **25 [58]**.

The ^1^H-NMR spectra of premyrsinane diterpenes generally exhibit signals attributed to four methyls (one secondary and three tertiary), four diastereotopic methylene groups, including one oxygenated at C-17, and three oxymethyne protons (H-3, H5, and H-7) (e.g., Compound **25**, Figure 7). Importantly, the presence of a *gem*-dimethyl-substituted cyclopropane ring, characteristic of these compounds but also of lathyrane diterpenes, is indicated by a pair of high-field methine signals at δ_H_ 0.72 (dd, H-11) and δ_H_ 0.82 (m, H-9), associated with the unusual up-field chemical shift of the quaternary carbon C-10 at δ_C_ 17.8. In addition to the acyl groups present in polyester diterpenes, the ^13^C spectra show twenty carbon resonances discriminated by the DEPT experiment as four methyl groups, three methylenes (one oxygenated at δ_C_ 69.9), nine methynes (three oxygenated at δ_C_ 81.5–65.8), and four quaternary carbons (two oxygenated at δ_C_ 86.6 and 90.2) [58].

**Figure 8:**
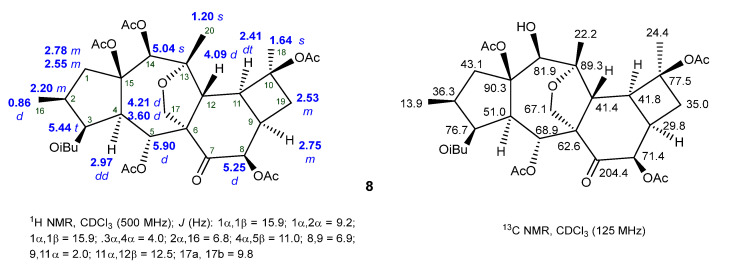
^1^H- and ^13^C-NMR spectroscopic data of the cyclomyrsinane diterpene **8** [51].

The NMR data of cyclomyrsinane diterpenes are very similar to premyrsinanes (Figure 7 and Figure 8). When comparing Compounds **25** and **8** [51,58], the most remarkable differences in the ^1^H-NMR spectra are the downfield shift of the H-9 and H-10 methine signals (δ_H_ 2.41 and 2.75) and the absence of one tertiary methyl signal (C-19) that is replaced by an additional diastereotopic methylene signal corresponding to H-19 in the cyclobutane ring. These data are corroborated by the ^13^C-NMR data, where the absence of the quaternary carbon at δ_C_ 17.8 and the presence of an additional methylene carbon (δ_C_ ~ 35.0) are very suggestive of a different D-ring on the molecule (a cyclobutane ring instead of a cyclopropane). In addition, the ^13^C-NMR data of Compound **8** (Figure 8) point to a carbonyl signal at δ_C_ 204.4 that, together with the downfield shift of C-6 (δ_C_ 62.6), indicate the presence of a ketone group at C-7 [51].

**Figure 9:**
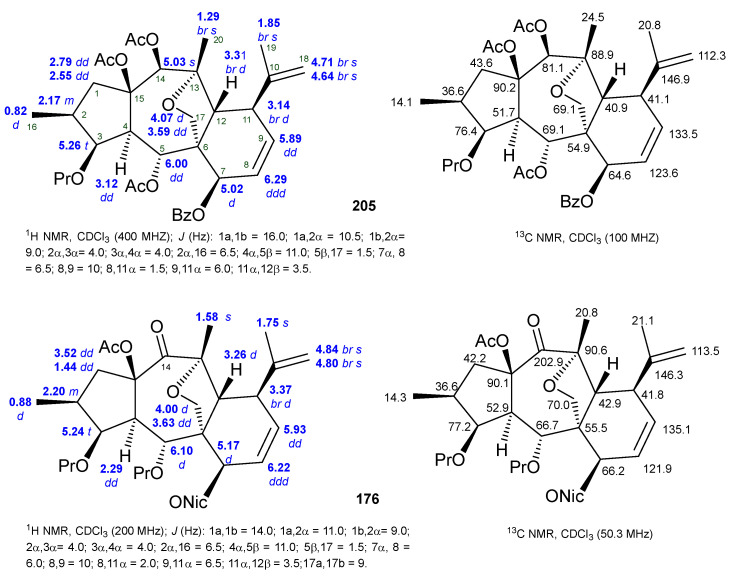
^1^H- and ^13^C-NMR spectroscopic data of myrsinane diterpenes **205** [37] and **176** [59].

By comparing the NMR data of premyrsinane and myrsinane diterpenes, it is evident that, except for the rings C and D, the ^1^H and ^13^C chemical shifts are almost identical (Figure 7 and Figure 9). Therefore, the most significant differences are, as expected, the absence of the cyclopropane signals and the additional existence of several NMR signals that are indicative of the myrsinane scaffold. In this way, the presence of the olefinic signals at δ_H_ 5.89 (δ_C_ 133.5) and δ_H_ 6.29 (δ_C_ 123.6) confirms the existence of the *vic*-disubstituted double bond (Δ^8,9^). The double bond Δ^10,18^ is deduced from the presence of two broad singlets assigned to the exocyclic methylene protons at δ_H_ 4.64 and δ_H_ 4.71 and corroborated by the vinylic methyl signal at δ_H_ 1.85, characteristic of the isopropenyl group (Compound **205**, Figure 9). The ^13^C NMR data support the presence of the two double bonds, revealing the additional resonances of the olefinic carbons (δ_C_ ~ 123.6, 133.5, 146.9, and 112.3). In some myrsinane diterpenes, the existence of a keto group at C-14 is common (Compound **176**, Figure 9) and deduced in the ^13^C NMR by the presence of the signal at δ_C_ 202.9 and the absence of one oxygenated methyne carbon. The location of the ketone at C-14 can be further supported by the down-field shift of H-1α (δ_H_ 3.52) and of the neighboring CH_3_-20 methyl group that appeared at δ_H_ 1.58 due to the anisotropic effect of the carbonyl group at C-14 when compared to the 14-acyl diterpene NMR data (Compound **205**, Figure 9) [37,59].

**Figure 10:**
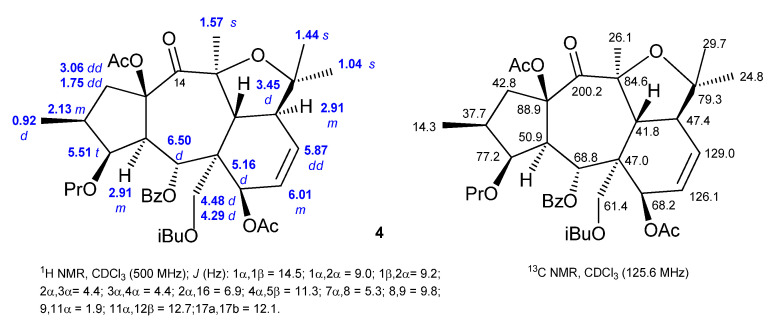
^1^H- and ^13^C-NMR spectroscopic data of rearranged myrsinane diterpene **4** [51].

Some rearranged myrsinane derivatives have also been isolated, possessing a tetrahydrofuran moiety between C-10 and C-13 (Figure 10). These compounds have a close resemblance to myrsinane diterpenes in most of their NMR features. Nevertheless, some differences are evident, namely, the replacement of the olefinic protons at C-18 by one tertiary methyl signal at δ 1.04 (s, H-18), the absence of the olefinic carbon signals at δ_C_ 146.3 (C-10) and δ_C_ 112.3 (C-18), and the appearance of a signal at δ_C_ 79.3, corresponding to the resonance of C-10 (Compound **4**, Figure 10) [51].

**Figure 11:**
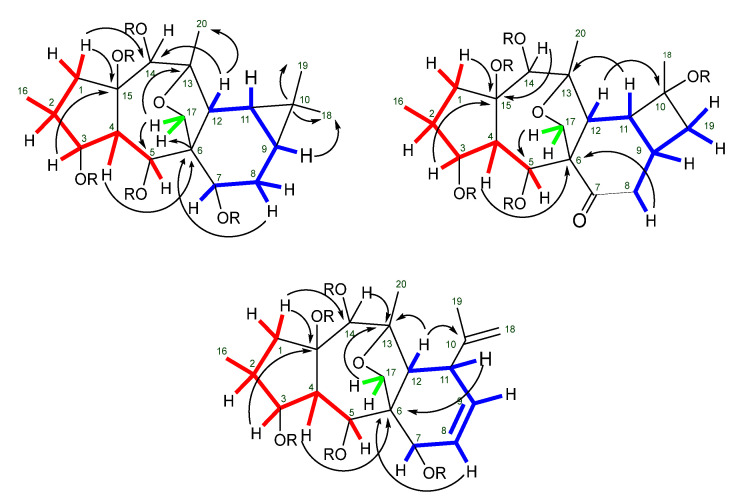
Selected ^1^H−^1^H COSY (bold, in red, green and blue) and HMBC (H→C) correlations for myrsinane, premyrsinane, and cyclomyrsinane skeleton.

The analysis of HMQC/HSQC and ^1^H-^1^H-COSY spectra allows the assignment of all the protons and carbon resonances and reveals the existence of three sequences of correlated protons, as depicted in Figure 11. The connectivities of these fragments are assigned by the heteronuclear ^2^*J*_C-H_ and ^3^*J*_C-H_ couplings displayed in the HMBC spectra between the quaternary carbons and the protons of the three spin systems, allowing the linkage of the referred fragments, the confirmation of the myrsinane-type scaffolds, and the location of the acyl moieties (Figure 11). Importantly, the HMBC correlations between the quaternary carbon C-13 and the protons H-17 and H-12 and between C-5 and H-12 and H-17 allow the assignment of the C-13/C-17 saturated furan ring, a very common feature in premyrsinane, myrsinane, and cyclomyrsinane diterpenes.

The relative configuration of the compounds is deduced from coupling constants analyses together with NOESY experiments, which establish the connectivities of the correlated protons through space. Based on biosynthetic reasons, the *trans* A-B ring fusion (Figure 2) and the α-oriented H-4, characteristic of macrocyclic diterpenes, are always taken as a reference point. In addition, for all the reported myrsinol-type diterpenes, Rings B and C (as well as Rings C and D in premyrsinanes and cyclomyrsinanes) are also *trans*-fused, and the diastereotopic methylene protons H-17 are α-oriented. In this way, the existence or absence of NOESY cross peaks between these key protons and the remaining protons or acyl groups allows the establishment of the stereochemistry of all the tetrahedral stereocenters of the molecule.

## 3. Distribution, Diversity, and Biological Activities of Myrsinane-Type Diterpenes from *Euphorbia* Species

Over recent decades, approximately 225 diterpenes have been isolated from several *Euphorbia* species. Herein, the distribution and the broad structural diversity of these diterpenes are summarized, also focusing on the reported biological activities. 

### 3.1. E. falcata

**Figure 12:**
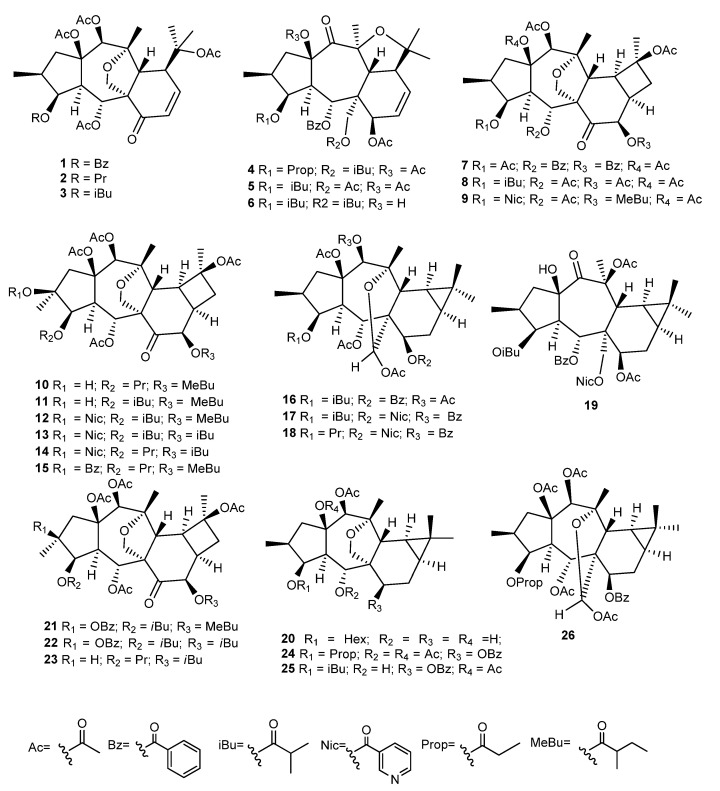
Chemical structures of myrsinane-type diterpenes isolated from *E. falcata*.

Several new myrsinane- (**1–6**), cyclomyrsinane- (**7–15**), and premyrsinane-type diterpenes (**16–19**) (Figure 12), named falcatins A–S, and a known cyclomyrsinane (**51**), named euphorprolitherin [60], were isolated from *E. falcata* methanolic extract [51,60]. These compounds, together with the known premyrsinane **20** and the cyclomyrsinanes **21–23** previously isolated from *E. prolifera* and *E. falcata* (Figure 12) [42,58,61], were studied for their GIRK and hERG channel-inhibitory activities, using an automated patch-clamp method [51]. The GIRK channels (G protein-activated inwardly rectifying potassium ion channels) are targets for the treatment of arrhythmias, as it was found that the selective inhibition of these channels reduces the number and the duration of atrial fibrillation episodes. On the other hand, hERG channels, also known as Kv11.1, are K^+^-selective voltage-gated ion channels, which mediate the rapid delayed rectifier K^+^ current (IKr) in ventricular myocytes. The hERG channels can be inhibited by different compounds, modifying the action potential of the heart muscle, increasing the risk of ventricular fibrillation and sudden cardiac death [51]. The possible potassium channel-blocking ability of Compounds **1**–**19**, **20**, and **21**–**23** has been evaluated. The GIRK channel inhibitory assay was performed on HEK-293 (human embryonic kidney) at two concentrations (1 and 10 μM). Twelve compounds (**1**–**3**, **8**, **9**, **12**, **14**–**15**, and **20**–**23**) were able to block the GIRK channel activity, with values ranging from 61 to 83% at 10 μM. In order to evaluate the selectivity of their GIRK blocking effect, further studies were performed on the HEK-hERG (human embryonic kidney cells) cell line at three concentrations (3, 10, and 30 μM). Among the tested compounds, falcatins A-C (**1**–**3**), H (**8**), and I (**9**) showed low inhibitory effects on the hERG channel and were, therefore, considered selective inhibitors of GIRK channels and potential lead compounds for the treatment of atrial fibrillation. Even though a detailed structure–activity relationship could not be established, the most promising compounds **1**–**3** have a myrsinane scaffold, with a carbonyl function at C-7, an ether bridge between C-17 and C-13, and no epoxy functionality between C-10 and C-13 [51].

Compounds **21**–**23**, together with premyrsinanes **20** and **24**–**26** previously isolated from this plant (Figure 12) [58], were evaluated for their antiproliferative activity against cervix adenocarcinoma (HeLa), endometrial adenocarcinoma (Ishikawa), and breast epithelial adenocarcinoma (MCF7) cell lines, using cisplatin as a positive control [61]. The tested diterpenes exhibited weak-to-moderate antiproliferative activity, with **25** being the most active, exhibiting a significant activity in all three cell lines (83.9% (HeLa), 93.6% (A431), and 59.2% (MCF7)), when tested at 30 μg/mL. The compounds were also evaluated for their ability to inhibit rhodamine 123 efflux in L5178 mouse lymphoma cells and the corresponding MDR sublines overexpressing MDR1 efflux pumps. On these sublines, where MDR1 is highly expressed, compounds with fluorescence activity ratio (FAR) values higher than 10 are regarded as strong modulators. Diterpenes **21**, **22**, and **24**–**26** were the most active, showing FAR values ranging from 52.6 to 74.5, when tested at 20 μM. Moreover, when tested at 2 μM, the compounds were also very active, exhibiting FAR values between 12.6 (**26**) and 46.1 (**21**) when compared with the positive control verapamil (FAR 8.7 at 22 μM). The type of in vitro interactions between the diterpenes and doxorubicin was also studied through combination assays on MDR mouse T-lymphoma cells. All compounds showed a synergistic effect with the anticancer drug. Compound **21** was considered of special interest for further studies since it was a strong modulator of the MDR pump and showed a strong synergism when tested in combination with doxorubicin [61].

### 3.2. E. prolifera Buch.-Ham

**Figure 13:**
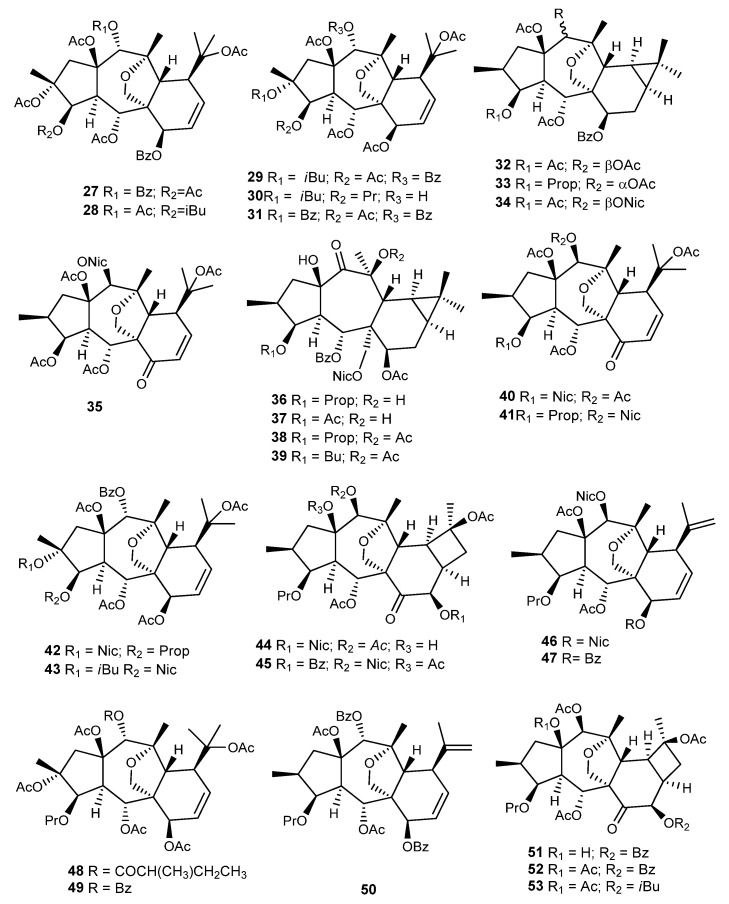

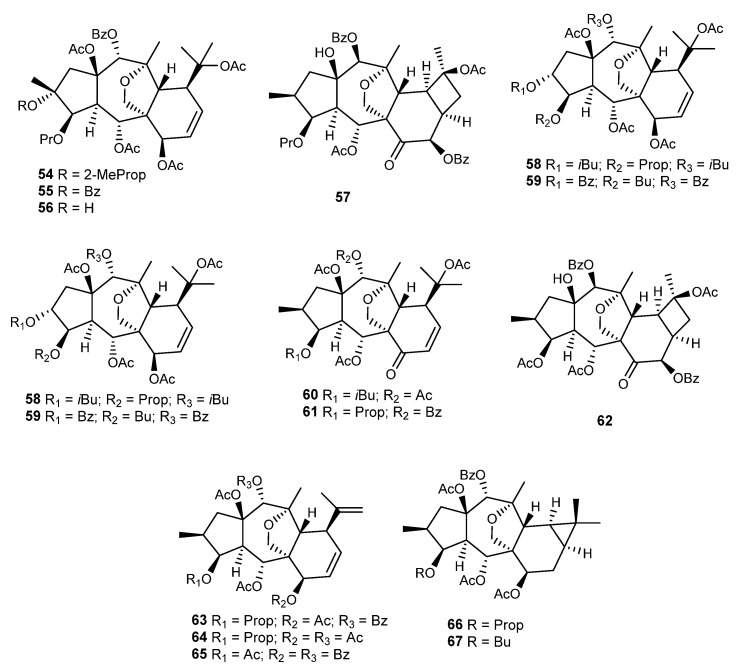
Chemical structures of myrsinane-type diterpenes isolated from *E. prolifera.*

*E. prolifera* Buch.-Ham. dried roots, known in China as Langdu, are a herbal medicine traditionally used to treat cancer and inflammation. The myrsinane diterpenes J196-10-1 (**27**) and J196-9-4 (**28**) (Figure 13) were isolated from the methanol extract and studied as possible modulators of Pgp in the human breast adenocarcinoma (MCF-7) cell line and its multidrug resistant subline (MCF-7/Adr) [62,63]. The structure of the compounds was very similar, only differing in the type of substituent at C-5 and C-14. It was found that both compounds were noncytotoxic to the tested cells at concentrations below 50 μM. When tested on the MCF-7/Adr cell line with daunorubicin, vincristine, and topotecan, the IC_50_ values of MCF-7/Adr to the anticancer drugs declined significantly. The best results were obtained for Compound **27** in combination with vincristine (IC_50_ = 0.063 ± 0.017 μM, reversal ratio value of 219.6, when coapplied with 10 μM of **27**). In the same experimental conditions, Compound **28** exhibited an IC_50_ value of 1.41 ± 0.054 μM. Further assays showed that both diterpenes inhibited the efflux of rhodamine-123 mediated by P-gp without changing the transcription level of ABCB1 gene, which suggested a direct inhibition of the drug efflux. Moreover, they were also able to stimulate ATPase activity at a low concentration, and it was suggested that they act as a competitive inhibitor of the enzyme. In this way, they might compete with cytotoxic drugs for Pgp binding sites, preventing the drug efflux [62,63].

Several diterpenes were also isolated from the roots of *E. prolifera* (**29**–**35**) (Figure 13) [64,65]. Compounds **29** and **30** were tested for their neuroprotective activity against MPP^+^-induced neuronal cell death in human dopaminergic neuroblastoma cells, using bakkenolide H as a positive control. Both compounds showed neuroprotective effects without exhibiting significant cytotoxicity [64].

Another phytochemical investigation of the roots of *E. prolifera* led to the isolation of ten new premyrsinol- (**36**–**39**), myrsinol- (**40**–**43**), and cyclomyrsinol-type (**44**–**45**) diterpenes, named euphorbialoids A–J, and two known analogues (**46** and **47**) (Figure 13) previously isolated from *E. seguieriana* [37]. All compounds were evaluated for their inhibitory activities on LPS-induced NO production in murine microglial BV-2 cells, using methyl-2-thiopseudouream sulfate as a positive control (IC_50_ 13.2 μM). Compounds **36**–**44** inhibited LPS-induced NO production in a dose dependent manner, with IC_50_ values ranging from 15.6 ± 1.5 μM (**37**) to 48.7 ± 1.9 μM (**44**). The compounds showed no cytotoxicity against BV-2 cells [66].

Zhang et al. isolated four new myrsinol diterpenes (euphorprolitherin A–D, **48**–**51**) (Figure 13). The structure and relative stereochemistry of euphorprolitherin A (**48**) was assigned using X-ray crystallography [60]. Together with these new derivatives, the cyclomyrsinanes SPr5 (**52**), previously found by Wu et al. in the same plant, as well as SPr1–4 (**53**), were also isolated, and the structures were confirmed using single-crystal X-ray analysis (Figure 13) [42].

Li et al. isolated four new myrsinane diterpenes (proliferins **A–D**, **54**–**57**) (Figure 13), with one being a cyclomyrsinane derivative (**57**) [67]. Proliferins A, B, and D (**54**, **55**, and **57**, respectively) were subjected to evaluation for cytotoxicity against HCT-8, Bel-7402, BGC-823, A549, and A2780 cancer cells. Only Compound **54** was found to be cytotoxic against A2780 human ovarian cancer cells (IC_50_ 7.7 µM). The other two compounds had no effect (IC_50_ > 10 µM) on any of the cell lines listed above [67]. In addition to the previously mentioned novel compounds, Li et al. [67] identified the known myrsinane diterpenes, namely, **207** [37], **49**, **51**, and **52** (Figure 13) [60].

In 2011, also from the roots of *E. prolifera*, Xu et al. isolated ten new myrsinane derivatives (**58**–**67**) [68] (Figure 13), together with nine known derivatives, namely, **205**, **206** [37], **137** [69], **49**, **50**, **52** [60], **54**, **55**, and **57** [67] (Figure 13). All the compounds exhibited neuroprotective effects against MPP^+^-induced neuronal cell death in SH-SY5Y cells, with Compounds **50**, **54**–**55**, **59**, **65**–**67**, **and 205** showing, at a concentration of 30 μM, a more effective activity than guanosine, the positive control used [68].

### 3.3. E. kopetdaghi (Prokh.) Prokh

**Figure 14:**
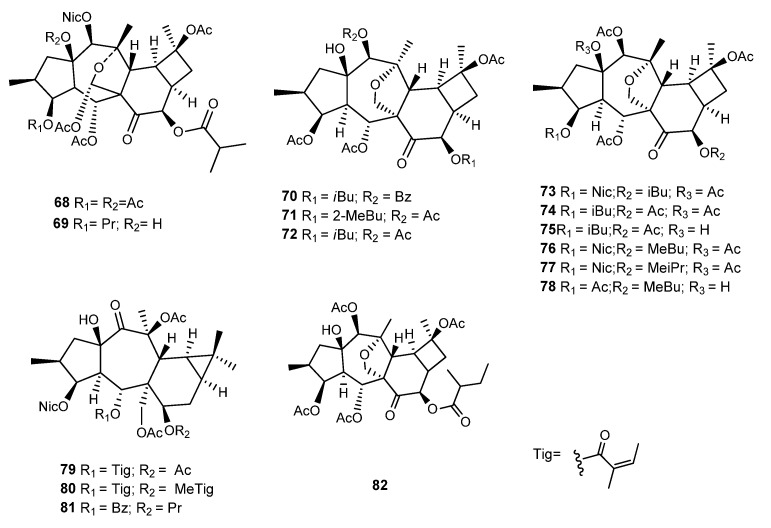
Chemical structures of myrsinane-type diterpenes isolated from *E. kopetdaghi and E. sogdiana*.

Riahi et al. reported two undescribed cyclomyrsinol diterpenes, **68** and **69**, named kopetdaghinane A and B (Figure 14), with an unusual 13/17 hemiacetal group in the tetrahydrofuran ring, isolated from the dichlorometane/acetone (2:1) extract of *E. kopetdaghi* (Prokh.) Prokh. aerial parts [70]. The cytotoxicity of **68** against MCF-7 and OVCAR-3 cancer cells was evaluated, and the compound showed dose-dependent activity, with IC_50_ values of 38.10 ± 2.05 μM and 51.23 ± 2.67 μM against MCF-7 and OCVAR-3 cancer cells, respectively. Moreover, this compound presents selectivity indexes against both cell lines of 18.36 ± 0.39 and 13.65 ± 0.29 against MCF-7 and OVCAR-3 cancer cells. Kopetdaghinane A (**68**) inhibits the growth of MCF- 7 breast cancer cells through the activation of the mitochondrial apoptotic pathway. The results of Western blot analysis showed that the expression of *Bcl-2* (an antiapoptotic protein) was remarkably decreased in response to treatment with **68**, whereas the expression of *Bax* protein (a proapoptotic protein) was increased. Regarding caspase-6 activity, the treatment of MCF-7 cells with an increasing concentration of **68** (1 to 100 μM) induced a marked increase in the activity of caspase-6 in a concentration- and time-dependent manner, with a depletion in mitochondria membrane potential (ΔΨm) [70]. 

From the acetone/chloroform (1:2) extract of the aerial parts of this species, three new cyclomyrsinol diterpenes were isolated [71]. Compound **70** (Figure 14) was noncytotoxic against the mouse embryo fibroblast cell line (3T3-L1) and rat Wistar hepatocyte cell lines (CC-1). Several assays were also carried out to study the immunomodulating activity of **70**, including a lymphocyte proliferation assay, the effect on interleukin-2 (IL-2) production, a phagocyte oxidative burst, and ROS production effects. It was found that **70** potently suppresses phytohemagglutinin-activated T-cell proliferation in a dose-dependent manner, with an IC_50_ value of 1.83 ± 0.15 μg/mL (Prednisolone was used as positive control, with an IC_50_ 1.45 ± 0.2 μg/mL). It also suppressed the IL-2 production, with an IC_50_ of 19.0 ± 0.7 μg/mL. The authors postulated that the simultaneous inhibition of T-cell proliferation and suppression of IL-2 production could suggest the possibility of interfering not only by downregulating IL-2 production but also directly with IL-2 function or even by the IL-2 deprivation-mediated apoptosis in vitro [71]. Compound **70** was also able to inhibit the production of reactive oxygen species (IC_50_ 1.54 ± 0.1 μg/mL), an effect that was suggested to be possible through ROS scavenging or by the inhibition of the enzymes (NADPH oxidase, catalase, and myeloperoxidase) involved in the signal transduction pathway of ROS generation process. The bioactivity of Compounds **71** and **72** (Figure 14) was not evaluated.

### 3.4. E. sogdiana Popov

Several cyclomyrsinane (**73**–**78**) and premyrsinane diterpenes (**79**–**81**) (Figure 14) were isolated from the acetone/dichlorometane extract of the aerial parts of *E. sogdiana* Popov [52]. The in vitro cytotoxic activity of the isolated diterpenes was screened against EJ-138 bladder carcinoma and Jurkat T-leukemia cell lines. Except for **77**, all the compounds were active against the Jurkat T-leukemia cell line, exhibiting IC_50_ values ranging from 11.3 μM (**81**) to 26.6 μM (**74**). Compounds **73**, **76**, and **77** differ in the type of acyl substituents at C-8, and it was found that the activity increases with the length of the acyl chain [52].

In a recent study, Rabbani et al. tested the cytotoxicity of six diterpenes, **72**–**74**, **76**, **78**, and **151**, previously isolated from *E. sodgiana, E. kopetdaghi*, and *E. aellenii* [52,71,72] on two breast cancer cells (4T1 and MCF-7) [73]. The results showed that **151** has the most cytotoxic effects against both cells, with IC_50_ values of 8 and 12 µg/mL for the MCF 7 and 4T1 cell lines, respectively. Furthermore, the cells treated with 5 and 10 µg/mL of Compound **151** for 24 h showed 37 and 55% of apoptotic cells. However, these cytotoxicity assays raise some doubts as a positive control was not used in this research, and, in addition, the results in µg/mL make it difficult to compare with data from other studies regarding cytotoxicity tests as normally these are given in µM.

A cyclomyrsinol named as TAMEC (**82**, Figure 14) [74] was evaluated on anxiety and depression-like behaviors as there is a significant prevalence of these disorders in people with multiple sclerosis (MS) [74]. The effects of TAMEC treatment on the progression of these clinical symptoms were studied in mice with experimental autoimmune encephalomyelitis (EAE) because it is the most widely used animal model to study multiple sclerosis (MS) at the research bench. This compound reduced anxiety and depression and reduced proinflammatory cytokines in the hippocampus of EAE-induced mice. More studies should be conducted to determine the involved histopathology and molecular mechanisms of TAMEC in modulating behavior in EAE-induced mice. 

### 3.5. E. gedrosiaca Rech.f., Aellen & Esfand

**Figure 15:**
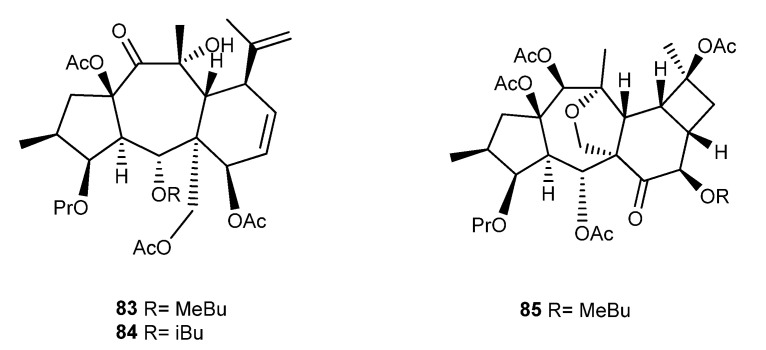
Chemical structures of myrsinane-type diterpenes isolated from *E. gedrosiaca*.

In a study on the composition of *E. gedrosiaca* Rech.f., Aellen & Esfand., Yazdiniapour et al. isolated and identified from a dichloromethane/acetone extract of the whole plant two new myrsinanes, **83** and **84** [54] (Figure 15), and two known cyclomyrsinanes, **53** and **85** (Figure 13 and Figure 15, respectively), previously reported from *E. prolifera* and *E. aellenii* [42,75]. The evaluation of their cytotoxicity against B16F10 and A375 melanoma cells indicated that, on both cell lines, these compounds showed cytotoxicity effects. According to the results, IC_50_ values for the treatment of melanoma cells by the new myrsinanes **83** and **84** were 58.45 and 55.43 μM on B16F10 and 20.66 and 21.88 μM on A375 cells, respectively. The cyclomyrsinanes **53** and **85** showed IC_50_ values of 86.52 and 82.27 μM on B16F10 and 36.21 and 39.87 μM on A375 cells, respectively. These findings demonstrated that Myrsinanes **83** and **84** clearly induce more cell death than Cyclomyrsinanes **53** and **85** [54]. 

A very recent study led to the isolation of four premyrsinane diterpenes and one myrsinane diterpene [76]. Premyrsinanes **120**, **131**, **132**, and **136** had previously been isolated from *E. sanctae-catharinae* [77] and *E. pithyusa* [69]. Myrsinane **49** has also been isolated from *E. prolifera* [60]. MDA-MB-231 and MCF-7, two cell lines associated with breast cancer, were used to assess the cytotoxic activity of these compounds, using the MTT viability assay. Compound **120**, with an IC_50_ value of 10.8 µM, was the most effective against both cell lines, particularly the MDA-MB-231 cell line. Premyrsinanes **131**, **132**, and **136** presented IC_50_ values of 24.5, 27.3, and 22.2 µM, respectively, against the MDA-MB-231 cell line. When tested for cytotoxicity against these two cell lines, Compound **49** showed less potency than the other four diterpenes. However, the IC_50_ value of 33.7 µM can be considered modest on the MDA-MB-231 cell line [76].

### 3.6. E. decipiens Boiss. & Buhse

**Figure 16:**
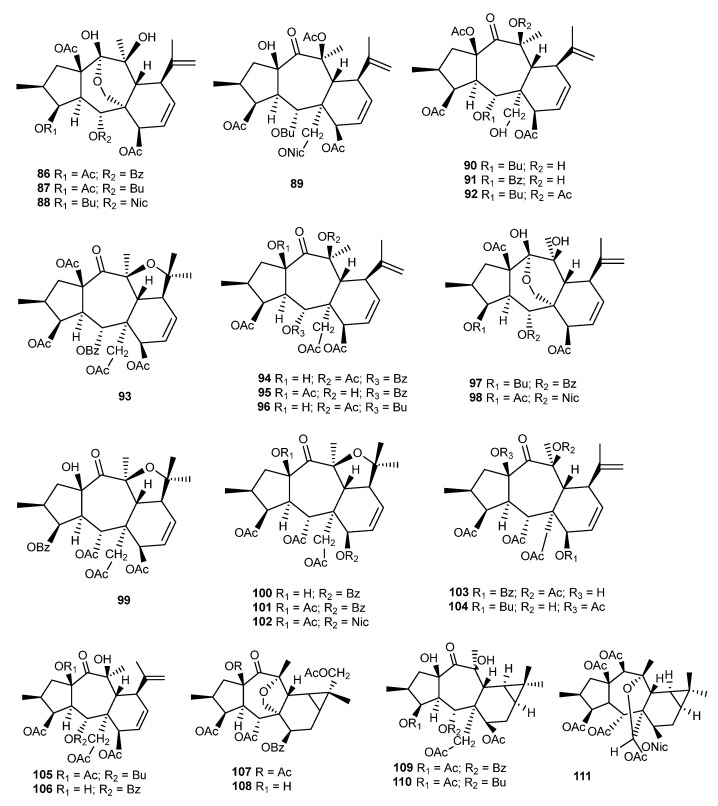
Chemical structures of myrsinane-type diterpenes isolated from *E. decipiens*.

From *E. decipiens*, whole plant several myrsinane diterpenoids were isolated during the last 30 years, namely, **86**–**88**, **89**, and **90**–**106**, together with five premyrsinanes (**107**–**111**) (Figure 16) [78,79,80,81,82,83,84,85,86]. Due to some technical problems in the structural characterization, mainly related to stereochemical errors, the structure of some of these compounds was corrected. For example, decipinones B (**86**) and C (**87**), which had previously been isolated and characterized by Ahmad and Jassbi [79], had their structure updated by Zahid et al., based on X-ray diffraction analyses [80]. The structure of decipinone A (**89**) also isolated by Ahmad and Jassbi presented here was the one described by the authors, and it probably had a wrong attribution [79]. On the other hand, the absolute configuration of decipinone (**94**) and isodecipinone (**95**), previously isolated and characterized by Ahmad et al. in 1998, was attributed later by Jassbi et al. [81,87]. 

Regarding the biological activity of myrsinanes isolated from *E. decipiens*, Compounds **97**, **99**, **100**, and **105** showed activity against prolyl endopeptidase [82,83,86], and Compounds **98**, **103**, **104** showed activity against the urease enzyme [82,84,88]. Compound **98** also showed a positive response to DNA-damaging activity in a mutant yeast bioassay [86], and Compounds **105** and **106** showed analgesic activity [83,85].

### 3.7. E. connata Boiss

**Figure 17:**
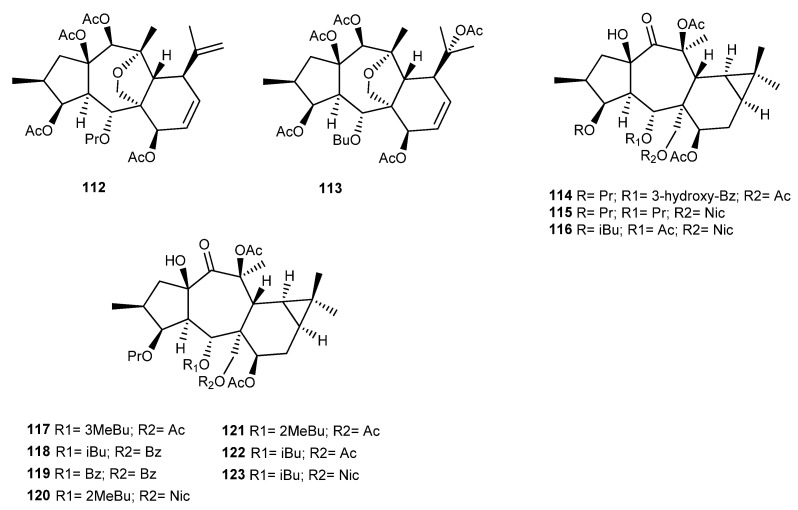
Chemical structures of myrsinane-type diterpenes isolated from *E. connata* and *E. sanctae-catharinae*.

Two myrsinane- type diterpenoids (**112** and **113**) were isolated from the acetone/chloroform (1:2) extract of *E. connata* (Figure 17). These compounds have been already isolated from *Pycnocycla spinosa* [55]. The compounds were screened for their cytotoxic activity against two human breast cancer cell lines (MCF-7 and MDA-MB468), showing IC_50_ values of 24.53 ± 3.39 and 26.67 ± 1.41 μM on the MDA-MB cell line and 37.73 ± 3.41 and 34.57 ± 2.12 μM on the MCF-7 cell line, respectively. Doxorubicin was assayed as the positive control (IC_50_ values of 0.16 ± 0.07 μM and 0.24 ± 0.08 μM on MCF-7 and MDA-MB cells, respectively) [56].

A recent study [89] aimed to explore the inhibitory effects of Compound **112** (named TPEM) on the growth of two human ovarian cancer cell lines, OVCAR-3 and Caov-4. The results obtained demonstrated that TPEM exerted inhibitory effects on these cells, dependent on the dose. The IC_50_ values found against OVCAR-3 and Caov-4 cells were 41.27 ± 1.52 and 36.44 ± 2.41 µM, respectively. In order to identify the molecular mechanism responsible for the observed cytotoxicity, the activity of caspase-3 was investigated, as this enzyme plays an essential role in the apoptotic signaling pathway. The results indicated that caspase-3 activity was notably increased by TPEM compared to the control group. In addition, **112** also exerts its cytotoxic activity by raising the level of ROS in cells [89].

### 3.8. E. sanctae-catharinae Fayed

From the aerial parts of *Euphorbia sanctae-catharinae* Fayed, an endemic species in Egypt, three new premyrsinane diterpenoids, namely, euphosantianane E–G (**114**–**116**), (Figure 17) were isolated [90]. Elshamy and collaborators performed an interesting correlation with the chemical composition of this endemic species of Egypt with other *Euphorbia* species through an agglomerative hierarchical clustering (AHC) analysis. *E. sanctae-catharinae* was grouped with *E. bupleuroides*, *E. fidjiana*, *E. fischeriana*, *E. pithyusa* subsp. *cupanii*, *E. prolifera*, and *E. seguieriana*. However, the Pearson correlation coefficient analysis revealed that *E. sanctae-catharinae* showed a close correlation to *E. bupleuroides* (0.888), followed by *E. prolifera* (0.880), and then *E. pithyusa* subsp. *cupanii* (0.870) based on the composition of the terpenoid composition. Myrsinol diterpenoids were the main constituents of these *Euphorbia* species, while other diterpenoid compounds such as abietane, lathyrane types, and others, as well as cycloartane triterpene, were also identified in these plants. In previous studies, these authors had already isolated from *Euphorbia sanctae-catharinae* nine premyrsinanes, (**117**–**123**) (Figure 17) four of which (**117**–**120**) were isolated for the first time, as well as previously reported metabolites that included three flavonoids [77]. Compounds **117**–**120** exhibited moderate antiproliferative activity against human cancer cell lines of colon (Caco-2) and lung (A549). Compounds **119** and **120** exhibited an IC_50_ value of 31.0 µM and 33.2 µM against Caco-2 cells and an IC_50_ value of 21.5 µM and 32.8 µM against A549 cells, respectively [90].

### 3.9. E. pithyusa L.

**Figure 18:**
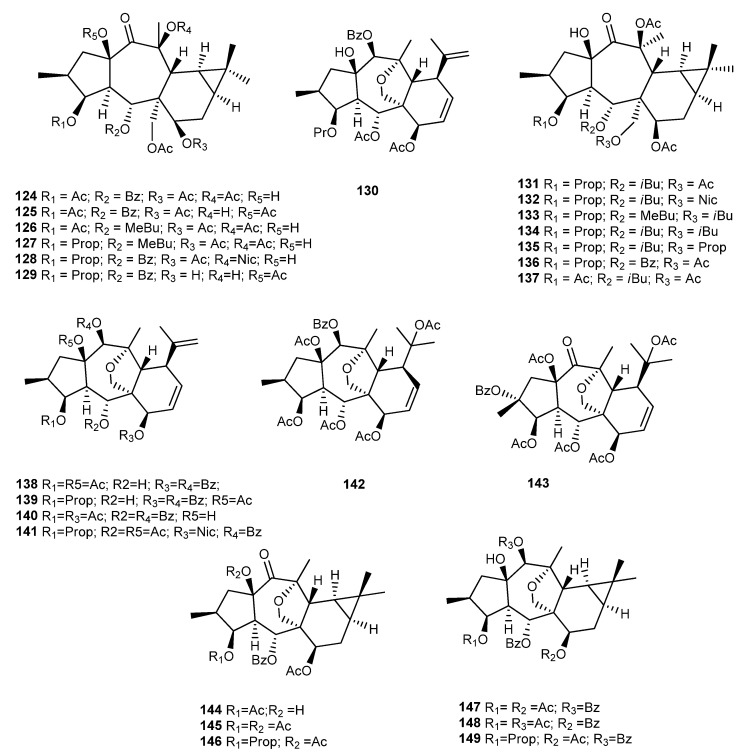
Chemical structures of myrsinane-type diterpenes isolated from *E. pithyusa and E. cupanii*.

In a previous study, nearly all EtOAc and MeOH extracts of eleven Euphorbiaceae species showed significant anti-chikungunya virus (CHIKV) activity, including *E. pithyusa* L. extracts [91]. Encouraged by these results, Esposito et al. isolated six new premyrsinanes (**124**–**129**) and one new myrsinane (**130**), together with a known compound (**136**) (Figure 18) and two known dideoxyphorbol esters from the aerial parts of *E. pithyusa* [92]. These compounds were evaluated against CHIKV replication, and from the tested compounds, the one that stands out in terms of anti CHIKV activity is the diterpene **129**, with an EC_50_ value of 11 ± 1.4 μM and a selectivity index of 5.8. The other premyrsinol esters (**124**–**128** and **236**) and the myrsinol ester **130** were not active (EC_50_ ≥ 50 μM and SI < 5). Interestingly, in the premyrsinol series, all compounds except Compound **130** have an ester group at C-7, suggesting that the acetate group at this location may be detrimental to antiviral activity [92]. Previously, Appendino et al. had already isolated seven new premyrsinane diterpenoids (**131**–**137**) (Figure 18) from the whole plant acetone extract of *E. pithyusa* subsp. Cupanii [69]. Among them, **136** was also isolated and identified by Xu et al. from *E. prolifera* Buch-Ham [68].

### 3.10. E. cupanii

The very close taxonomic relationship between *E. cupanii* and *E. pithyusa* led Esposito and collaborators to investigate the diterpene ester content of *E. cupanii* using LC-MS/MS [57]. Molecular networking coupled with unsupervised substructure annotation (MS2LDA) indicated the presence of new premyrsinane/myrsinane diterpene esters in *E. cupanii* extract fractions, which was confirmed by the isolation of six new myrsinanes (**138**–**143**) and six new premyrsinanes (**144**–**149**) (Figure 18), along with other known diterpenes, namely, phorbol esters. In conclusion, the profile of *E. cupanii* is marked by myrsinol and 13,17-oxy-premyrsinol esters. As several phorboids isolated from *Euphorbia* spp. were shown to inhibit chikungunya virus (CHIKV) replication, the authors in this research only evaluated these compounds for their ability to inhibit CHIKV replication and did not evaluate myrsinanes and premyrsinanes [57].

### 3.11. E. aellenii Rech

**Figure 19:**
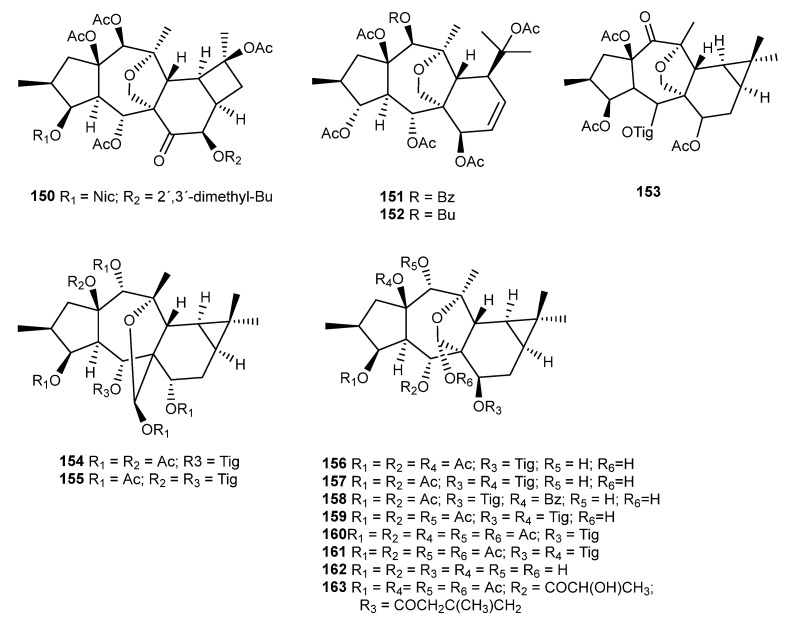
Chemical structures of myrsinanes isolated from *E. aellenii* and *E. aleppica*.

The immune system is an important target for the development of treatment strategies to improve the management of infections. Moreover, it has been known for many years that the modulation of the immune response is possible using compounds isolated from plants. Two new (**150** and **85** Figure 15 and Figure 19, respectively)) and one known (**200**) cyclomyrsinane-type diterpenes were isolated from *E. aellenii* Rech. f. [75,93]. In vitro immunomodulatory activity was assessed by measuring the phytohemagglutinin (PHA)-induced T-cell proliferation. All compounds showed low inhibitory activity (0–23%) when tested at 0.5 µg/mL; however, the effect increased when tested at higher concentrations, with inhibition values ranging from 15% to 29% (at 5 µg/mL) and 38% to 53% (at 50 µg/mL). The most active compound was **200**, which exhibited an IC_50_ value of 40.4 ± 9.35 µg/mL. 

A previous phytochemical investigation of the chloroform fraction of the methanol extract of the aerial parts afforded two 14-desoxo-10, 18-dihydromyrsinane diterpenoids (**151** and **152**, Figure 19), which were tested for their immunomodulatory effect. Compound **151** showed an antiproliferative effect on lymphocytes but also the capacity to inhibit the zymosan-induced oxidative burst in whole blood phagocytes (up to 50%) at a concentration of less than 0.5 µg/mL [72].

### 3.12. E. aleppica L.

A study of new cytotoxic diterpenes of the premysinane type from *E. aleppica* L. was conducted by Zolfaghari et al. [53]. A new compound **153** and a previously isolated compound **154** [94] (Figure 19) were tested against MCF-7 and MDA-MB 231 breast cancer cells. Compound **153** showed moderate cytotoxicity, but Compound **154** exhibited an higher dose-dependent cytotoxic effect, with IC_50_ values of 17.6 ± 1.2 and 16.7 ± 1.5 µM against MCF-7 and MDA-MB 231, respectively. The authors suggested that the cytotoxic activity is probably due to the presence of 14-O-acetyl instead of the ketone group at C-14 and 17-O-acetylated hemiacetal instead of the 13(17)-epoxy ring in Compound **153**. In addition to **154**, several diterpenoids of the premyrsinane type with different substituted patterns had already been found when extracts of acetone and petroleum ether/Et_2_O/MeOH (1:1:1) were studied (**155**–**163**) [38,40,41,94].

### 3.13. E. lathyris L.

**Figure 20:**
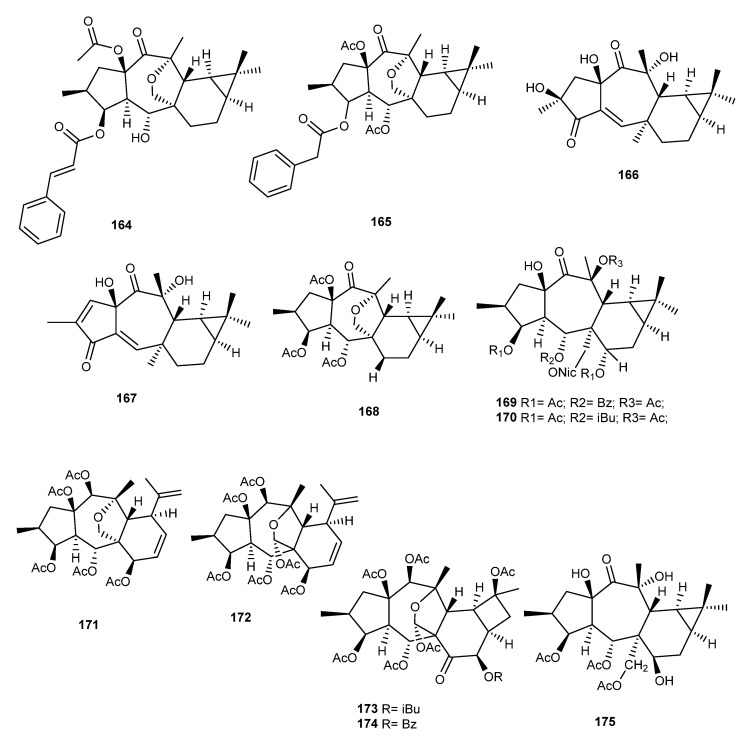
Chemical structures of myrsinane-type diterpenes isolated from *E. lathyris*, *E. macrorrhiza* and *E. boetica*.

From the seeds of *E. lathyris* L., Qian Wang et al. found, for the first time, a new diterpenoid of the premysinane type, called premylanin (**164**) (Figure 20), along with thirteen known compounds and four new diterpenoids of the latyrane type. The cytotoxicity of the isolated compounds against the cancer cell lines HCT116, MCF-7, 786–0, and HepG2 was evaluated, but **164** exhibited little inhibitory activity at 50 µM for 24 h [95].

In a more recent study, Compound **165** (Figure 20), a novel premyrsinane diterpenoid, and other diterpenes with various skeleton types were also isolated from the seeds [96].

### 3.14. E. macrorrhiza C.A. Mey 

From the acetone extract of the whole plant of *E. macrorrhiza* C.A. Mey., several macrocyclic and rearranged macrocyclic diterpenes were isolated, including a new premyrsinane, macroripremyrsinone A (**166**), and jatrophodione A (**167**), previously isolated from *Jatropha curcas* [47] (Figure 20). Compound **166** was shown to be noncytotoxic against the human oral epidermoid carcinoma (KB) cell line and its navelbine-selected ABCB1 overexpressing (KBv200) cell line (IC_50_ > 50 μM), whereas **167** exhibited IC_50_ values of 22.46 ± 2.72 μM and 47.87 ± 0.30 μM for the KB and KBv200 cell lines, respectively. Compound **167** was also tested in combination with the anticancer drug navelbine for its ability to modulate multidrug resistance on the KBv200 cell line which overexpresses P-gp and exhibited MDR reversal activity (IC_50_ 0.37 ± 0.013 μM) [97].

### 3.15. E. boetica Boiss

From the methanol extract of the aerial parts of *E. boetica* Boiss., three new premyrsinanes (**168**–**170**), two new myrsinanes (**171**, **172**), and three new cyclomyrsinanes (**173**, **174**) (Figure 20), along with three known diterpenes, belonging to the cyclomyrsinane and lathyrane types, were isolated **[50]**. In this study, only the lathyranes were evaluated for their ability to promote the proliferation of neural precursor cells (NPCs) [50]. Also, from *E. boetica*, a new premyrsinol derivative without the ether linkage between C-17 and C -13, named eufoboetol-3,5,17-triacetate (**175**) (Figure 20), was isolated and characterized [98].

### 3.16. Other Species

**Figure 21:**
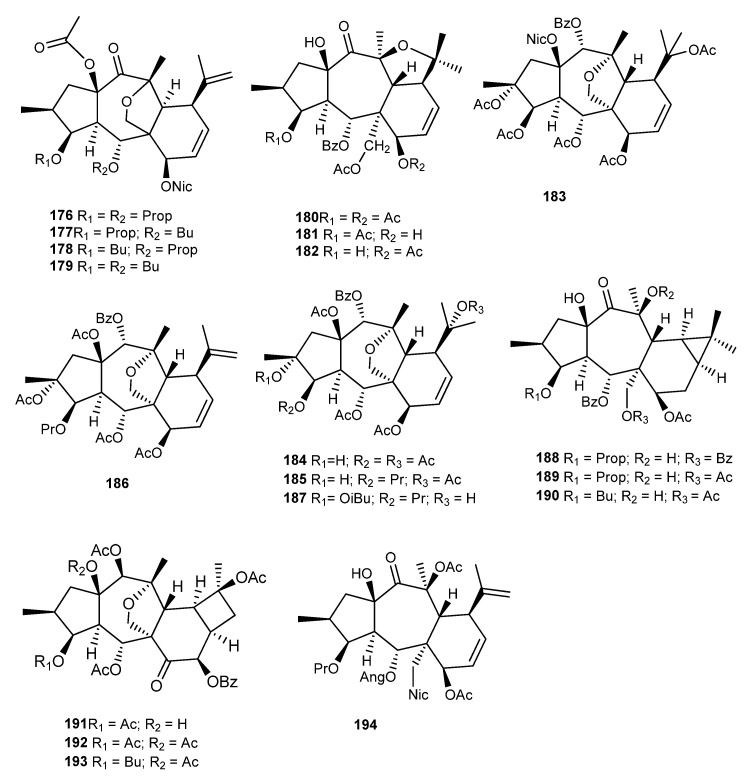
Chemical structures of myrsinane-type diterpenes isolated from *E. myrsinites, E. cheiradenia, E. dracunculoides*, and *E. erythradenia*.

From the roots and aerial parts of *E. myrsinites* L., four myrsinane-type derivatives, **176**–**179** (Figure 21), were isolated and characterized by Öksüz et al. [59]. Compounds **178** and **179** showed a moderate anti HIV-1 reverse transcriptase (RT) inhibition activity, with IC_50_ values of 80 and 67 µg/mL, respectively, and **176** and **177** were inactive (IC_50_ > 200 µg/mL) [59]. Compounds **177** and **179** were also isolated from *E. marschalliana* by Jassbi et al., which confirmed their stereochemistry through the ROESY NMR experiment. The authors corrected the stereochemistry at C-6, C-12, and C-13 [99].

In 2000, Abbas et al. isolated from the acetone extract of aerial parts of *E. cheiradenia* Boiss. & Hohen. three myrsinane derivatives with an unusual tetrahydrofuran moiety due to the epoxy bridge between C -10 and C-13, namely, cheiradone (**180**), cheiradone A (**181**), and cheiradone B (**182**) (Figure 21). Among them, cheiradone (**180**) showed inhibitory activity against *α*-glucosidase enzyme type V1, with an IC_50_ value of 0.32 mM [100]. Later, in a study conducted by Hussain et al., cheiradone (**180**) also showed to inhibit VEGF-induced angiogenesis by binding to VEGF receptors −1 and −2 [101].

From the 70% aqueous Me_2_CO extract of *E. dracunculoides* Lam., several new myrsinane- (**183**, **184**–**187**) [102,103,104], premyrsinane- (**188**–**190**) [105], and cyclomyrsinane-type (**191**–**193**) (Figure 21) [105] diterpenes were isolated, together with known compounds that were previously obtained from other *Euphorbia* species [37,66,67]. Also, from *E. erythradenia* Bioss., a new myrsinane was isolated **194** (Figure 21) but without biological activity unlike the tetrahydroingenol diterpenes isolated from this plant [106].

**Figure 22:**
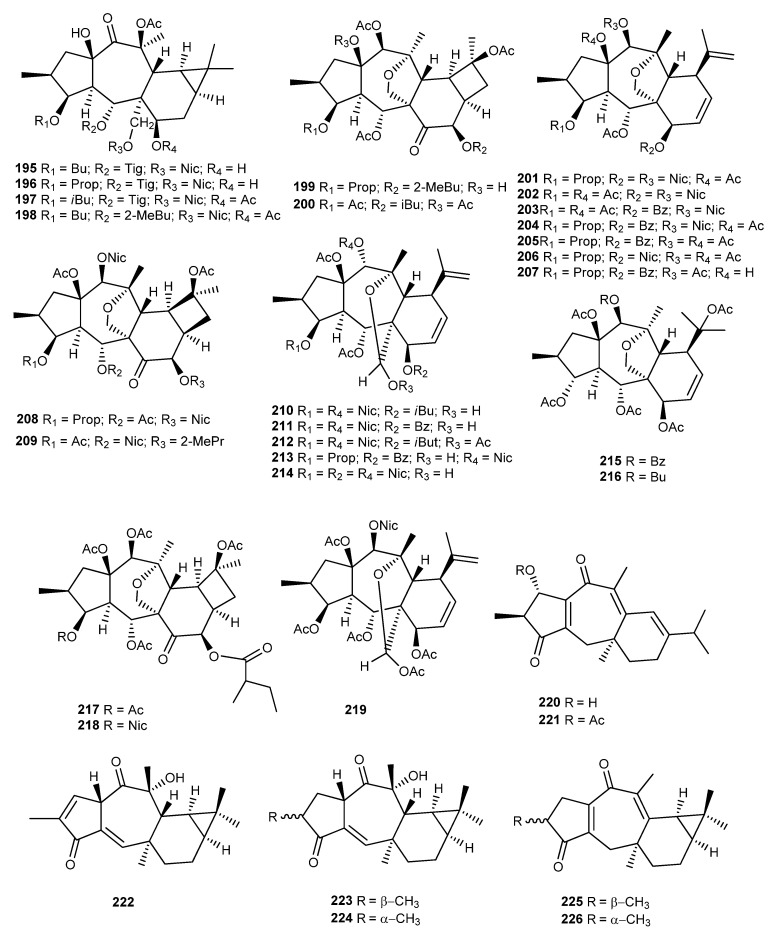
Chemical structures of myrsinane-type diterpenes isolated from *E. macroclada*, *E. microsciadia*, *E. seguieriana*, *E. splendida*, *E. teheranica*, and *Jatropha curcas*.

Four premyrsinane diterpenoids (**195**–**198**) (Figure 22) were isolated from aerial parts of *E. macroclada* Boiss. by Shokoohinia et al. [107].

Ghanadian et al. isolated two new cyclomyrsinol esters (**199** and **200**) (Figure 22) from the methanol extract of *E. microsciadia* Boiss. aerial parts [93]. Compound **199** was tested for its in vitro antiangiogenic activity on vascular endothelium growth factor in cultured human umbilical vein endothelial cells, showing 23.9 ± 2.6% inhibition at the lower concentration of 0.5 μg/mL. In addition, the antiangiogenic effect was correlated with the concentration, increasing at 5 μg/mL to 34.6 ± 2.7% and further increasing at 50 μg /mL to 42.1 ± 0.8%. 

Jeske et al. isolated from the whole *E. seguieriana* var. seguieriana Neck. plant seven myrsinane-type diterpenes (**201**–**207**) (Figure 22) and a cyclomyrsinane derivative, **208** [37]. Another cyclomyrsinol diterpene derivative (**209**) was identified by Öksüz et al. from the acetone extract of *E. seguieriana*, together with five new 17-hydroximyrsinane diterpenoids (**210**–**214**) (Figure 22) [108].

From whole plant *E. splendida* Mobayen methanol extract, Ayatollahi et al. isolated a known compound decipinone (**94**) (Figure 16), which was first isolated from *E. decipiens* [109].

Two diterpenoid esters of the cyclomyrsinane type (**217**–**218**) and one 17-hydroximyrsinane derivative (**219**) were isolated from *E. teheranica* Boiss. (Figure 22) [110].

Bao et al. isolated jatrocurcadiones A (**220**) and B (**221**) (Figure 22), two novel diterpenes with an unusual 10,11-seco-premyrsinane skeleton, from *Jatropha curcas* L. twigs [49]. These compounds are the first example of the cleavage of C10/C11 bond of the cyclopropane ring in the premyrsinanes. Their biogenetic pathway is summarized in Figure 5. Compound **220** was tested as an inhibitor of thioredoxin reductase, a major regulator of the intracellular redox homeostasis, whose deactivation is believed to be implicated in the inhibition of the proliferation and induction of necrosis or apoptosis. The diterpene exhibited potent inhibitory activity, with an IC_50_ value at 10.0 µM, was more active than the positive control curcumin (IC_50_ 25.0 µM) [49].

The phytochemical study of the air-dried roots of *Jatropha curcas* cv. *nigroviensrugosus* extract resulted in the isolation of four premyrsinane diterpenes, named jatrophodiones B–E (**223**–**226**) [48] (Figure 22), which were structurally similar to jatrophodione A (**222**), previously isolated from the same species by Xu et al. [47]. Jatrophodiones D (**225**) and E (**226**) were isolated as an inseparable mixture of isomers, only differing in the stereochemical centre at C-3. These compounds were evaluated for cytotoxicity against HL-60, SMMC-772, A-549, MCF-7, and SW480 human tumor cell lines but were inactive in this test (IC_50_ > 40 µM) [48].

## 4. Conclusions

Over the past four decades, more than 200 myrsinane-type diterpenes have been isolated from *Euphorbia* species. It is interesting to note that the presence of these type of compounds are restricted to only around 20 species, of which *E. falcata*, *E. prolifera*, *E. kopetdaghi*, and *E. decipiens* stand out. In this way, they can be regarded as potential chemotaxonomic markers of some *Euphorbia* species due to of their delimited distribution and structural diversity. 

The biosynthesis of myrsinane diterpenes is still not fully understood, particularly the mechanistic details on the conversion of the lathyrane skeleton to premyrsinane and its derivative counterparts. Herein, we discussed the possible biosynthetic pathways and chemical conversion of premyrsinanes to cyclomyrsinanes and myrsinanes. Furthermore, some relevant information about the isolation procedures and structural identification of representative myrsinane-type diterpenes, using NMR spectroscopy, is also presented for the first time in a schematic and comprehensive manner. It is our hope that the summarized key spectroscopic features will help researchers to identify myrsinane-type diterpenes in a faster and more straightforward manner. 

Most of the research data published in the literature have described the isolation and structural identification of new myrsinane polyesters and reported some biological activities that are summarized in Table 1. The most frequently performed experiments were aimed at exploring the cytotoxic potential against several cancer cell lines, the modulation of multidrug resistance, immunomodulatory and neuroprotective effects, and the inhibition of enzymes such as urease, HIV-1 reverse transcriptase, and prolyl endopeptidase. However, when compared to other *Euphorbia* diterpenes, such as lathyranes and jatrophanes, that have been extensively investigated, the biological activity of myrsinane-type diterpenes is still much less studied, and these compounds were, in general, less bioactive. In addition, in the majority of the studies, the mechanisms of action have not been explored nor has the structure–activity relationships been established, probably due to the reduced amount of the isolated compounds, which is always an important drawback in these studies. For the same reason, there are currently no reported in vivo or toxicological studies.

Nevertheless, the data gathered in this review encompass the academic works and efforts of several research teams across the globe, and, in our opinion, it is quite pertinent. Through a chemotaxonomic approach, it can be used to identify other promising species to investigate, as plants belonging to the same genus or family should produce similar specific secondary metabolites. Moreover, these outcomes would provide researchers with new insights to more easily identify these types of structures and compare biological activities with previously identified myrsinane-type diterpenoids, which can help to find possible therapeutic application for these compounds.

## Figures and Tables

**Figure 3:**
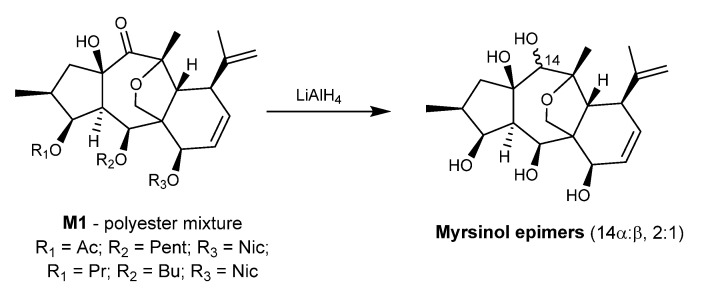
Reduction of the polyester mixture obtained from *E. myrsinitis* to obtain the 14α,β myrsinol epimers [39].

**Table 1:** Summary of the main biological activities reported in the literature for myrsinane-type diterpenes isolated from *Euphorbia* species.

***Euphorbia*** ** spp.** **Extract (Plant Material)**	**Compounds**	**Biological Activity**	**Ref.**
***E. aellenii***			
CHF/ACET (aerial parts)	**200**	Compound **200** showed in vitro immunomodulatory activity (IC_50_ 40.4 ± 9.35 µg/mL) and a significant inhibition of lymphocyte proliferation at 50 µg/mL.	[75]
MeOH (whole plant)	**151**	Compound **151** exhibited immunomodulatory properties by inhibiting the zymosan-induced oxidative burst in whole blood phagocytes (up to 50%) at a concentration of less than 0.5 μg/mL.	[72]
*E. aleppica*			
DCM/ACET (2:1)	**154**	Compound **154** exhibited cytotoxic activity against two breast cancer cell lines (IC_50_ 17.6 ± 1.2 and 16.7 ± 1.5 µM, MCF-7 and MDA-MB231, respectively).	[53]
*E. connata*			
ACET (aerial parts)	**112**, **113**	Moderate cytotoxicity against several cancer cell lines (IC_50_ between 24.5 and 37.7 µM). The mechanism responsible for the observed cytotoxic effect was related to an increase in caspase-3 activity; Compound **112** also raised the ROS levels in cancer cells.	[56,89]
*E. decipiens*			
ACET (whole plant)	**97**, **99**, **100**, **105**	Inhibitory activity against prolyl endopeptidase.	[82,83,86]
	**98**, **103**, **104**	Inhibition of urease activity; Compound **98** showed a positive response to DNA-damaging activity.	[82,84,86,88]
ACET:CHF (whole plant)	**106**	Inhibitory activity against prolyl endopeptidase and analgesic activity.	[83,85]
CHF (whole plant)	**105**, **106**	Significant analgesic activity when administered to mice at dose of 5–20 mg/kg i.p. (This activity is comparable to that of 100 mg/kg of aspirin or ibuprofen.)	[85]
*E. falcata*			
MeOH (whole fresh plant)	**1–3**, **8**, **9**, **12**, **14**, **15**, **20–26**	Targets for the treatment of arrhythmia by blocking GIRK channel activity, with values ranging from 61 to 83% at 10 µM; Compounds **1**–**3**, **8**, and **9** were considered selective inhibitors of GIRK channel. Compound **25** exhibited antiproliferative activity against HeLa (83%), A431 (93.6%), and MCF7 (59.2%) cell lines when tested at 30 μg/mL.	[51,61]
		Compounds **21**, **22**, and **24–26** were strong modulators of MDR1 efflux pump on MDR mouse T-lymphoma cells (FAR values between 52.6 and 74.5 when tested at 20 μM). Compound **21** was also very active when tested at 2 µM (FAR 46.1) and displayed a synergistic effect with doxorubicine.	
*E. gedrosiaca*			
ACET:DCM (2:1) (whole plant)	**53**, **83–85**	Cell growth inhibitory activity and apoptotic effects on melanoma cell lines (B16F10 and A375); **83** exhibited IC_50_ 20.66 μM on A375 cells.	[54]
(aerial parts)	**49**, **120**, **131**, **132**, **136**	Cytotoxic effects in a dose-dependent manner against breast cancer cell lines (MDA-MB-231 and MCF-7); Compound **120** exhibited an IC_50_ 10.8 μM against MDA-MB-231 cells.	[76]
*E. kopetdaghi*			
ACET:CHF (1:2) (aerial parts)	**70**	Compound **70** was evaluated through lymphocyte proliferation assay (IC_50_ 1.83 µg/mL), IL-2 assay (IC_50_ 19.0 µg/mL), oxidative burst of phagocytic leukocytes (IC_50_ 1.6 µg/mL). No cytotoxicity was observed against two cell lines (CC-1 rat hepatocyte and 3T3-L1 mouse fibroblast cell lines)	[71]
ACET:DCM (1:2) (aerial parts)	**68**	Compound **68** showed cytotoxicity against MCF-7 (human breast cancer cells) and OCVAR-3 (human ovarian cancer *cells)*, with IC_50_ values of 38.10 ± 2.05, and 51.23 ± 2.67 μM, respectively, with a good selectivity index for cancer cells lines. Decrease in Bcl-2 expression, the increase in Bax protein expression, and the induction of caspase-6 activity could explain its cytotoxic effect.	[70]
*E. macrorrhiza*			
ACET (whole plant)	**167**	Compound **167** showed cytotoxic activity against KB cell lines (IC_50_ 22.46 ± 2.72) and MDR reversal activity when tested on MDR-resistant KBv200 cell line (IC_50_ 0.37 ± 0.013).	[97]
*E. microsciadia*			
MeOH (aerial flowering parts)	**199**	Compound **199** inhibited VEGF-induced tube-like structures by 23.9 ± 2.6% at the lower concentration of 0.5 µg/mL. (VEGF activity may be modulated by varying its concentration.)	[93]
*E. myrsinites*			
EtOAc (aerial parts)	**176**–**179**	Moderate inhibitory activity for HIV-1 reverse transcriptase; compounds were tested for anti-inflammatory activity but did not exhibit significant activity.	[59]
*E. pithyusa*			
EtOAc(aerial parts)	**130**	Compounds **130** exhibited significant inhibiting activity against CHIKV replication, with EC_50_ value of 11 ± 1.4 μM (SI 5.8)	[92]
*E. prolifera*			
MeOH (dried roots)	**27**, **28**	Compounds **27** and **28** were strong modulators of P-gp expressed in MCF-7 cell line, with IC_50_ values of 0.063 and 1.41 µM, respectively; **27** exhibited a reverse ratio value of 219.6 when in combination with vincristine and inhibited the efflux of rhodamine-123 mediated by P-gp, without changing the transcription level of ABCB1 gene as Compound **28**. Compounds **27** and **28** stimulated ATPase activity at low concentrations, and it was suggested they act as competitive inhibitors.	[62,63]
	**29**, **30**	Neuroprotective effects against MPP+-induced neuronal cell death in SH-SY5Y cells without cytotoxic effects.	[64]
	**36**–**44**	Inhibitory activities on LPS-induced NO production in murine microglial BV-2 cells without cytotoxic effects.	[66]
	**50**, **54, 55, 59, 65–69, 205**	Exhibited a more effective neuroprotective effect against MPP^+^ -induced neuronal cell death in SH-SY5Y cells than guanosine used as positive control.	[68]
EtOH 95% (roots)	**54**	Compound **54** showed cytotoxicity against human ovarian cancer cell line (A2780), with an IC_50_ value of 7.7 µM.	[67]
*E. santae catharinae*			
DCM:MeOH (1:1) (aerial parts)	**117–120**	Moderate antiproliferative activity against A549 and Caco-2 tumor cell lines; Compound **119** exhibited IC_50_ values of 31.0 µM (Caco-2) and IC_50_ 21.5 µM (A549); Compound **120** exhibited IC_50_ values of 33.2 µM (Caco-2) and 32.8 µM (A549).	[77]
*E. sogdiana*			
ACET:DCM (2:1) (aerial parts)	**73**–**78, 79**–**81**	Moderate cytotoxicity against bladder carcinoma (EJ- 138) and Jurkat T-leukemia cell lines (IC_50_ values between 11.3 and 26.6 μM).	[52]
	**72–74, 76**, **78,151**	Cytotoxic effects against breast cancer cell lines (4T1 and MCF-7), with Compound **151** being the most cytotoxic (8 and 12 µg/mL, respectively).	[73]

Extracts: ACET—acetone; CHF—chloroform; DCM—dichloromethane; EtOH—ethanol; EtOAc—ethyl acetate; MeOH—methanol.
